# Housing first for middle aged and older women: the emerging case

**DOI:** 10.3389/fgwh.2025.1726756

**Published:** 2026-01-13

**Authors:** Joanne Bretherton

**Affiliations:** School for Business and Society, University of York, York, United Kingdom

**Keywords:** domestic abuse, homelessness, housing first, violence, women

## Abstract

This paper explores the use of Housing First services for women experiencing homelessness, focusing on those aged 35 and over, who have multiple and complex needs. The paper draws on an evidence review and the results of a five-year evaluation of a Housing First for Women pilot project (2015–20) and three-year longitudinal study of two further Housing First services for Women in the UK (2021–24), which centred on the lived experience of women using these services. Four main arguments are advanced. The first is that the original Housing First model from the US and the initial deployments of the Housing First approach in Europe and the UK used a model designed in a context in which the nature and extent of middle aged and older women's homelessness was poorly understood. High fidelity Housing First services were less likely to be fully effective because the original model did not properly account for the level of trauma associated with domestic abuse and violence against women in middle age and later life. The second argument is that there is, on current and emergent evidence, a clear case for developing Housing First that is designed, managed and run by women for women which includes safeguarding as one of its key operating principles. The third argument is that Housing First for Women, with its comprehensive co-productive support and intensive case management, may offer important advantages over Sanctuary Schemes ^1^ and other services that are designed to counteract middle aged and older women's homelessness that is associated with abuse, violence and multiple and complex needs. The paper concludes by arguing that in order to fully meet the needs of middle aged and older women experiencing long term and repeated homelessness with multiple and complex needs, an integrated and preventative strategy, including preventative approaches like Domestic Abuse Housing Alliance (DAHA) Accreditation and Housing First for Women must be developed. If Housing First for Women is to be effective, it must be situated within a wider integrated strategy to counteract women's homelessness to reach its full potential.

## The emergence of housing first

Much of the research on homelessness done in the last 40 years has been focused on a disproportionately lone male population, often in early to late middle age, with multiple and complex needs, who experience long term or repeated homelessness (1, 2). Housing First, a US programme model originally developed in the early 1990s, was designed specifically for people experiencing this form of homelessness (3, 4).

Housing First grew out of a systemic problem with US responses to this form of homelessness, which had centred on linear residential treatment or 'staircase' programmes. Staircase programmes used an institutional model to bring someone to a state of housing readiness by completing a series of (programme required) steps. At each step, behavioural issues and treatment needs around addiction and mental illness would be addressed, using compulsory support and treatment, within a framework of (re) conditioning someone to maintain their own housing more or less independently (5). Attrition from staircase services was high, with between 40 and 60 percent of service users either being ejected from programmes before their homelessness was resolved, or choosing to self-exit, which was a high failure rate given these services were relatively expensive (6).

Housing First was a radical departure from staircase services (3). Housing was provided to people experiencing homelessness who had multiple and complex needs immediately, or at least very quickly. There was no requirement that an individual complete a number of 'steps', show behavioural change and be treatment compliant before they were offered a home. The Housing First approach was not passive, it still sought to create changes that would end the risk of a return to homelessness, but to do so within a collaborative, co-productive “consumer choice” model which worked with service users. Housing was not conditional on working with a Housing First programme, i.e., housing and support were separated, which meant someone kept their housing even if they stopped using the support on offer. Housing First also used a harm reduction approach, unlike the staircase services which tended to rely on abstinence and detoxification programmes. Unlike many previous homelessness programme models, Housing First was also designed to be open ended offering support for as long as was needed. Rather than seeking to make someone housing ready before offering housing, Housing First quickly placed them in their own home and then used intensive case management and multi-disciplinary teams to work with that person to develop the support, treatment and other help they needed to permanently exit homelessness (4).

Importantly, Housing First was designed to be holistic, multi-agency response that addressed other needs once secure housing was in place. The intensive case management (ICM) at the core of the service was designed to meet treatment and support needs around addiction, mental health, physical health, personal (social) care, access to welfare benefits, friendship and social networks, family networks (where appropriate) and engagement as a citizen and a neighbour in the community and wider civic and economic life (3). The original model also employed an assertive community team (ACT) approach for those with the very highest needs, in essence incorporating an interdisciplinary team within Housing First, which meant less reliance on case management and joint working with health, social care (social work), mental health, addiction and other services. However, in practice the costs of the ACT approach means it is less widespread than Housing First which employs only ICM in the UK and Europe (7, 8).

Beyond some successes with pilot projects and programmes in individual cities and US States (4), the use of a Housing First model with US veterans who were experiencing homelessness associated with multiple and complex needs proved particularly successful (9). Outside the US, experimental trials of Housing First conducted in Canada and France showed the Housing First model was relatively more effective than existing services (10), with very similar results being reported by other European countries that also began experimenting with Housing First (7).

Praise for the Housing First approach was not uniform, with some critics arguing that while it was more successful in ending homelessness, its co-productive/consumer led approach to treatment and support meant that outcomes were more uneven that programmes that required compulsory treatment (11). The political (far) Right has always disliked Housing First as it emphasises both structural causes of homelessness, i.e., that society is unequal, unfair and fails to adequately support highly vulnerable, economically marginalised people and advocates a structural solution, i.e., providing housing and support, rather than pathologizing homelessness (12). The Trump administration attempted to ban Federal funding of Housing First in July 2025 which may have had a severe impact by the point this paper is available. However, the successes that Housing First has achieved in reducing homelessness among people with multiple and complex needs have often overwhelmed critical voices and Housing First programmes have tended to multiply, especially in Europe (4, 13).

The progress made by Finland, which had developed its own particular version of what is also termed Housing First as the core of an integrated national strategy (14), began to draw international attention in the mid 2010s. Unlike most other European countries, overall levels of homelessness and particularly long term and repeated homelessness among people with multiple and complex needs began to fall, with Finnish successes outpacing those in similar countries such as Denmark and Ireland (15). While distinct from the original American ideas of Housing First, Finnish strategy and service models shared a lot of ideas around enabling choice and control for people experiencing homelessness, harm reduction and using a housing-led, rather than staircase approach (16).

## Housing first for women

Women's homelessness tends to be undercounted. One reason is that administrative separation of domestic abuse and violence against women and girls (VAWG) services from homelessness systems, has meant that statistical data often records rates of abuse and violence, but not the homelessness that results from it. Another reason is that the statistics on “family” homelessness tends to conceal the evidence that the bulk of homeless families containing dependent children are headed by a lone woman parent. Finally, there is growing evidence that women, including women with multiple and complex needs who are experiencing repeated and sustained homelessness, are often in situations of “hidden” homelessness living precariously with friends, acquaintances and sometimes family in 'sofa surfing' arrangements (17–21).

Women's homelessness also appears to be distinct from that of men because of the strength of the associations between the causation and experience of women's homelessness, domestic abuse and VAWG. Much of the available evidence indicates that experience of violence and abuse by women experiencing homelessness is nearly *universal* i.e., the rates are at such levels that women who are homeless not reporting these experiences are the exception and that this pattern is global, at least across the developed economies (17, 21–26).

Experiencing long-term homelessness (27) and indeed homelessness in general (28, 29) may influence the effective rate at which someone ages. The prevalence of non-communicable disease, limiting illness and disability is at very high levels among people experiencing longer term homelessness (30, 31). To express this in slightly oversimplified terms, a fifty-year-old woman experiencing sustained or repeated homelessness, is likely to present with morbidity closer to that of a housed woman twenty or thirty years her senior, i.e., at fifty, her physical health is likely to look like that of a seventy or eighty year old.

The core argument, for which there is some evidence, is that experiencing homelessness for long periods of time appears to effectively accelerate the physiological effects of ageing and to do so significantly (29, 32). Controlling exactly for the effects of homelessness on women's health is difficult, poorer women tend to be generally unhealthier, and to develop non-communicable disease earlier, than richer women and deeply socially unequal societies are unhealthier than those with better levels of equality (33, 34). People experiencing homelessness are, almost universally, from (very) poor backgrounds (35) which means their health is likely to be relatively poor even before they become homeless.

Early mortality is another pronounced characteristic of populations experiencing homelessness. Homelessness does not exist in one form and nor is it defined in consistent ways (2) and so rates of mortality do vary across populations. The most extreme examples of early mortality tend to be found among people sleeping rough (experiencing street homelessness), with one recent study reporting a median age of 51 (36). There is also evidence that people with sustained experience of homelessness tend to die decades earlier than the general population (37, 38). Living into later life, in the sense of first reaching retirement age and then living well into your seventies, is uncommon among people with sustained or recurrent experience of homelessness. There also risks of early mortality linked to addiction, intravenous drug use and blood borne infection (39), although this is more prevalent among some groups of people experiencing homelessness than others (38).

Visible homelessness among women, revealed in surveys and administrative statistics, has been on the increase for some time, including among women with multiple and complex needs who are in middle age and later life (17, 40). People using Housing First tend towards middle age, as they have developed multiple and complex needs over time, which is linked to a wider ageing of the long-term and repeatedly homeless population in North America (27, 32). Women's homelessness is undercounted (21), however, when the RCT of the Canadian Housing First programme reported initial findings, the fact that the service users averaged 39 years old was not a surprise, but the report that 32% of them were women was still a little unexpected (41). In the later French Housing First RCT, there was again a preponderance of middle age (an average of 39) service users of whom 17% were women (10).

Arriving at an archetype of a woman using a European or North American Housing First service is a little difficult, as there are no administrative records at national level on homelessness service use, in most countries, with some exceptions including Denmark and Ireland (15). However, the wider evidence base on Housing First suggests a woman in early to late middle age, who is likely to be a parent but also be separated from her children, who is *very* likely to have experienced domestic abuse and to present with addiction, diagnosed mental illness, limiting illness or disability and some history of criminality (42, 43). There is also strong evidence of early mortality across Housing First service users, with Housing First programmes routinely taking on a palliative role for their typically middle to late middle aged users (44–46) albeit with some research suggesting higher rates of excess mortality in men (47).

The case for Housing First for Women (HFW) rests on two main arguments. The first is that the prevalence of trauma, which has mainly resulted from male-perpetrated VAWG makes anything other than a Housing First service designed, built and delivered entirely by women an impractical prospect. The second is that women with multiple and complex needs, experiencing recurrent and sustained homelessness, who again are within the population for which Housing First is intended, have needs that are often clearly distinct from those of men within that same population (20, 48). Whereas social isolation and a lack of social connection and networks often characterise men who use Housing First, women may often be at ongoing risk of abuse and violence and require safeguarding as a major element of the support that HFW provides (42, 43, 49–51).

Research specifically on HFW is still underdeveloped. In 2023, O'Campo et al. (52) noted that there was “scant research on the effectiveness of supported housing for women” (52). This paper draws on two UK case studies: the results of a five-year evaluation of a HFW pilot project (2015–20) and a three-year longitudinal study of two further HFW projects (2021–24), both of which centred on the lived experience of women using HFW. The main goal of the paper is to critically evaluate the suitability of this emerging form of Housing First for middle-aged and older women, which is already receiving both national and international attention as a new form of Housing First, with guidance being offered by the Housing First Hub Europe.^2^

## Methods

The first study looked at a pilot programme HFW service over the course of five years. The pilot worked with 41 women experiencing long-term and repeated homelessness over the course of the research, most of whom had had contact with the criminal justice system. Women using this HFW service tended to be in their 30 s and 37% were aged over 35. Sixteen women who were using this service were interviewed over the duration of the research (42). The second study looked at another dedicated HFW service and a second attached model, in which an existing Housing First service added specific female-only worker support for women using an existing service. In effect, this second service provided a second strand of HFW service alongside a wider Housing First programme. Collectively, these two services from the second study worked with 24 women over the course of the evaluation of whom nine were interviewed over the three-year evaluation of these services. Almost all the respondents in this second study were aged between 35 and 55 (43). In total the three HFW services worked with 65 women of whom 25 were interviewed over the five and three year periods of their respective evaluations. One caveat to note here is that information on age was deliberately imprecise, as participation was anonymous for the women using HFW who opted to take part. This was in part to give the women confidence that nothing personal would ever be shared about their lives and because ethical approval required that the GDPR legal framework governing special category data^3^ in the UK had to be followed, as the interviews might include the women talking about experiences of abuse, violence, drug use, criminal activity and other sensitive subjects. These ethical and legal requirements meant that for the women participating age ranges (e.g., 30–34) rather than birthdays, or exact ages, were recorded. Five younger respondents (i.e., thought to be under 35) have not been included in the analysis within this paper.

Interviews ranged in length from between twenty-five minutes to over an hour. Collectively, the interviews represented some 90 thousand words from women talking about their experiences of using HFW.

Both studies used a semi-structured topic guide that centred on women's lived experience of using the HFW service. Women were encouraged and supported to talk freely about their lives, their concerns, experiences and the strengths and weaknesses of the HFW service they were receiving, with the interviewers ensuring the key points of the evaluation were covered but otherwise working to enable the women to express themselves as freely and openly as they were comfortable with. This approach meant that interviews did not have a planned length, as the discussion was largely led by the women deciding on what they wanted to talk about and how much they wanted to say about each subject. Interviews might be as short as twenty five minutes, or they might go on for longer than an hour. Thematic grid analysis was employed to look systematically at the interviews, with the research teams on each of the two studies cross checking their analyses and interpretation to ensure that the results were being interpreted consistently.

Before fieldwork began on both studies full ethical approval was applied for and received by the [details to be added]. This adhered to the University's Code of Practice and Principles for Good Ethical Governance.

The results reported here focus on women using HFW who were in middle age or older. Again, the paper focuses on the lived experience of women using HFW focusing on the qualitative data from the semi-structured interviews.

## Results

The results across both studies echoed both the wider evidence base on women's experience of homelessness (21) and the emerging international evidence base on HFW (50, 51). The women using the two HFW services had near universal experience of violence and abuse. Their other needs centred on very high rates of addiction and a high prevalence of (diagnosed) mental illness, with rates of limiting illness and disability that were far above those for housed women of a similar age profile. The women who participated in the interviews were, as the wider evidence base suggests, frequently parents who had either placed their children with other relatives, such as grandparents, when homelessness threatened or seen their children taken into care because of issues with addiction, criminality and, particularly, mental health.

It's only, what, two years ago. Two years. I was selling drugs. I used to rob people for money.

God, eight years, I think, now. I first went down, because handing my kids over, I had a nervous breakdown. I was full-blown on drugs, go out in prostitution.

Perhaps the most significant finding across the three HFW services was the extent to which women reported that HFW was different from the other forms of homelessness service they had used. The three points that were emphasised were the empathy and understanding of the women who provided the HFW services they were using, the flexibility of the support on offer and the ways in which HFW had provided them with what they viewed as sustainable exits from homelessness. These responses from four of the women using the HFW services summarise these findings.

Because I'm a sceptical person. I just thought they were a bunch of do-gooders who like to preach at you and actually get no help along the way. The outcome: they've helped me a lot. They don't push it…they supported me through everything.

And somebody's who's got your back as well and sees your side of things and your point of view, cos like the social workers are there for the kids and they don't care about like how the mother's feeling or anything, whereas these workers are there for the kids and the mother.

And it's like what I've found with other services…they would judge me… say I can't work with you..whereas this service is completely different, the workers actually listen to me, and they ask me what was wrong as to why I was saying them things and why I was behaving in that way, and they talked me through, and they talked me down to where I was calm and laughing again. So just cos [because] they had listened to me and spoken to me about it.

Yeah, massive with, massive with the tenancy, getting me the tenancy, setting me up with the, the basic things; now and again they've had to get me food from the, what do they call them? Food banks. And just talking, just talking and knowing it's safe to talk.

There was strong evidence that the HFW could provide an effective and sustainable exit from homelessness for women with multiple and complex needs. Once housing was secured, both the efforts that the HFW services put into securing appropriate homes and making sure those homes were suitable were praised by many women using Housing First. There were also some instances in which HFW could quickly secure the right housing. These positive experiences can be summarised in comments by three of the women using HFW services.

I got a flat within a few weeks. It was […] brilliant, it really was. The time and effort they put in. I just thought, yes, they just want to help me, and they'll put me somewhere in some shithole and it was lovely. It really was. They did it up for me and it made me feel hopeful that they could be there for me. They made it homely; you name it. They were there constantly, every few days, or every week.

Housing First did all my decorating, yes. They decorated for me, yes. Yes, I told them what colours I wanted, and they decorated it all for me. It were lovely. They had the furniture in with it as well, yes.

I feel like I'm making a nice home, it's not just a place, it's a home, and it's mine, I can call it mine, so that's a nice feeling as well, to say that I've got my own home.

Flexibility of response in the support offered, reflecting the wider emphasis on a consumer-led (co-productive) approach, which was evident in all three HFW services was also seen in a positive light. This reflected the points that women using HFW made about the way in which HFW workers listened to them and respected and reflected the women's views in the support they offered. This ability of HFW to respond flexibly was also seen as providing comprehensive help that reflected particular sets of needs. The views of four women using HFW provide an overview of this wider experience.

I phone up [the worker] with a problem. I have no way of sorting this out, but [HFW worker] says, ‘Calm down, give me 2 min, I’ll sort it out’, she phones back, and it is sorted and I'm like, ‘Wow’, the big weight has gone off my shoulders, and I'm not stressed anymore for the whole day, otherwise I would be stressing for the whole week until I saw her… It's made me a happier person, definitely..

It's because it's settled, I'm happy, and I'm actually working with [HFW] workers, I have been for a while now. I think once you start involving in service more, getting involved with them, and taking the support what they're giving you, your life settles down a lot more. You get into a routine. It works having a routine.

[HFW women workers] Make sure I get to my doctors' appointments, get me to the hospital if I need to, tell me if I'm getting too far gone. They know me. They know if I'm letting myself go; they try and advise me if I need to go to, ‘Come on, [respondent name], I think it might be time to get you to hospital'…I nearly died, I think, a couple of years ago. If it wasn't for them taking me there, I wouldn't have gone, so yes. I think they actually saved my life

Yes, obviously it's got better—a lot better—because I am stable and I'm secure. No one can take that away from me; that's nice so that would be itself. But then I've had low points as well because I've had addiction and stuff that I've dealt with. Where I've not had medication, my mental health has been really bad, but it's getting—I'm in a lot better place now, so yes, it's got better.

The challenges that could face women in HFW and the teams of HFW workers who were helping them could however be considerable. Women using HFW were still often at risk from former partners (in all instances, male) who had perpetrated violence and abuse against them. In most instances, the HFW services had provided sufficient security and distance, while also ensuring information on women's whereabouts was kept confidential. However, a few women were still found and the HFW services would sometimes have no alternative but to move them. The women who were using HFW who are the focus of this paper i.e., those in mid to late middle age tended to have the high rates of poor health, limiting illness and disability that characterise women who have experienced repeated or sustained homelessness. In essence, their poor physical health compounded their potential vulnerability and the HFW services had all encountered examples of “cuckooing” in which someone or some group of people forced control over a woman's flat (apartment) or house (53). Again, HFW could react by involving the Police, housing managers and landlords, but sometimes had little realistic option other than to move the women out of risky and dangerous situations. These occurrences were not typical of most women's experiences using HFW services, but they were again indicative of the particular challenges that a HFW service faces, and the necessary emphasis on safeguarding that differentiates it from the original Housing First model (3). Two women using HFW provide an overview of the kinds of risks and challenges that could sometimes be faced.

I got cuckooed, as you call it. Yes, got cuckooed, as you call it… So, they moved me out quick, which were good.

This [second home provided by HFW service] is better as it has got a front and a back door, the first one only had a front door and he used to stand in front of it, so I had no exit..so I didn't feel particularly safe in that place… and I've got security doors now as well, so I feel a lot safer.

Another set of challenges that HFW services could face centred on access to treatment, support and other public services. Much has been written on the barriers that can be faced by people experiencing homelessness when trying to access social services (adult social care), general practitioners (family doctors) and outpatient services, as well as psychiatric and mental health social work services and addiction services (31), including some analysis of the specific barriers for women (15, 54). HFW is designed to counteract the barriers that women with experience of homelessness can face to health and care services, such as loss of medical records, a long history without a fixed address and attitudinal barriers, that include sexism and misogyny (55), through the use of intensive case management. However, in a context of continued retrenchment of the UK state from health and social services, a situation of general scarcity and long waits these services, experienced by the general public as well as by the women using HFW, was an operational challenge for HFW. Access to a psychiatrist or an addiction service might be arranged by a HFW service, but if the waiting list was months long, that service became effectively unavailable, hampering HFW services as they tried to assemble the right mix of treatment and support (42, 43).

Meeting housing need at the speed at which had been intended in the original Housing First programmes was very challenging for all three of the HFW services. All three HFW services were working in highly commodified housing markets where both rents and mortgage costs were hyperinflated and after housing cost poverty was widespread, reflecting the wider, longstanding position across the UK and Europe (56). Waits for affordable and settled (rented) housing in the private rented and social rented sector [approximately 16% of UK housing stock is social housing, see: (57)] in temporary accommodation could be weeks or months. Once suitable housing was secured the results were generally good, but the sometimes very long waits could be very challenging, particularly if women using HFW were temporarily accommodated in places that were not of a very good standard or in areas in which they did not feel safe.

As is the case with Housing First in general (45, 58), HFW has some operational limits in the extent to which it is able to address the multiple and complex needs of the women it is designed to support. The first point here, which has been used as a way to attack the Housing First model (12), is that women using HFW arrive with often very poor mental and physical health, with experience of domestic violence and abuse and other traumatic experiences and high rates of addiction, which means recovery is unlikely to be rapid, nor will it necessarily be complete (45).

The women using HFW did not report rapid gains in mental and physical health, or sudden shifts in addictive behaviour, but rather a pattern of slow, sometimes uneven progress. Even if a HFW service was unable to access all the support service and treatment services a woman needed, or was only able to secure housing that was not ideal or had some problems, then outcomes around health, wellbeing, mental health, addiction, access to social support and community participation could be uneven.

As the women using HFW moved into later middle age, increased limiting illness and disability would often appear, creating issues with mobility and their capacity to do some day-to-day activities. HFW services could seek to arrange adult social care services [UK terminology for personal care, e.g., help with dressing, washing, toileting or feeding, provided by social (work) services] and medical assistance from the NHS (National Health Service, the UK universal public health system), but could not directly help with these care and treatment needs. Equally, when women using HFW required support or treatment around mental health and addiction, the services could seek that help on their behalf, but had only relatively limited capacity to intervene directly.

I have no intention of going to work. I can't even walk up a set of stairs. I can't carry big boxes. My attitude is quite… I'm a bit funny—not with people; I can chat to anyone, but as working in a shop or something that'd do my head in, that… God, I couldn't handle that. I suffer anxiety. I was shaking. I'd be depressed, I might say the wrong thing and I can’t help that..

I don't think I would've got out of it without the support of [HFW worker], the support of here. I don't think I would because I got really ill. I've got palindromic arthritis as well so I'm on hydroxychloroquine for that. But when I first got that, I was smoking and the pain was just horrific and I didn't know what was going on with me. I weighed seven-and-a-half stone.

There are operational limits to HFW. These limits appear when treatment and support needs either become sufficiently acute to mean that close cooperation with adult social care and NHS services becomes essential to maintaining a woman with experience of homelessness in her own home and, if such cooperation is not present, HFW will find itself operating beyond safe capacity. Alongside this, women using HFW services may reach a point where limiting illness, disability, mental health or other treatment and support needs means they should no longer be living independently in the community because the risk is too high, even where support from health and adult social care is in place.

## Discussion

Drawing a clear line between the needs and experiences of women in middle and later life and the needs of younger women using HFW programmes does present some challenges. One issue here is what might be termed the (effectively) accelerated ageing that can accompany recurrent and sustained homelessness, so that a woman in her twenties or early thirties with multiple and complex needs may present with treatment and support needs that are not prevalent in housed populations of their age, but which are more common in women who are twenty or thirty years older (29–32). Perhaps the most striking finding of this research and the other evidence that is starting to be gathered on HFW is the level and complexity of need with which women of all ages were presenting. Addiction, severe mental illness, trauma associated with domestic violence and abuse, separation (quite often forced) from children, limiting illness and disability were all highly prevalent and the likelihood of highly complex and multiple needs appeared to broadly increase with age. In essence, all the women using HFW had had very damaging and traumatic experiences and suffered from poor mental and physical health and as they advanced in years, the intensity, range and scope of those treatment and support needs tended to increase (42, 43).

Other research on long-term and repeated women's homelessness has reported this same broad picture, i.e., high and complex needs throughout the population, which are exacerbated as age—and hence the duration of homelessness—increase (59–62). This finding suggests that HFW should be an integral part of wider strategy to meet the needs of women in middle age and older women experiencing repeated and sustained homelessness. This said, it must be noted that both the evidence bases on the nature and extent of women's homelessness (21) and around how many older people are experiencing homelessness and what their needs are (63) remain relatively underdeveloped. This links a wider point that, with some exceptions like Denmark and Ireland and to some extent the UK, data on the nature and extent of homelessness are incomplete in many developed economies and definitions of what constitutes homelessness are also inconsistent (64).

More evidence is needed on the exact scale of women's homelessness in middle age and later life, as well as their needs, characteristics, experiences and trajectories through homelessness, particularly repeated and sustained homelessness (21). Nevertheless, the work presented here and other research on HFW shows this population does exist and does have multiple and complex needs, and that HFW may well be part of a potentially effective response to their needs (42, 43, 50, 51).

By offering holistic case management support that works with the women using these services, HFW for women can address treatment needs, support needs and offer direct practical and emotional support to women experiencing homelessness who have multiple and complex needs. There is also evidence here of a great complexity and depth of treatment and support needs for which only a service model like HFW, which is able to react flexibly and comprehensively, is suitable. Protection from domestic abuse and VAWG, long centred on women's refuges (shelters) and more recent models like Sanctuary Schemes, enabling women to keep their homes by managing ongoing risk from perpetrators offer safeguarding, but do not offer the support provided by HFW (65–67).

While progress has been made in preventing homelessness triggered by domestic abuse and VAWG by the Domestic Abuse Housing Alliance (DAHA)^4^ model in the UK (68), this is an “early warning” and intervention model and again does not offer the support for women with multiple and complex needs offered by HFW alongside safeguarding. There is no logistical reason why, with the correct safeguarding in place, that HFW cannot be used in a preventative role, enabling a woman with multiple and complex needs who is at risk to retain her existing housing. The original Housing First model allowed for this kind of preventative intervention, i.e., stepping in where a high risk of homelessness linked to individual needs, characteristics, experiences and situation (3). Another emergent variant of Housing First, Housing First for Youth (HF4Y) is explicitly targeted on an at-risk group of young people with multiple and complex needs who have experience of child protection systems. This can also be used as a preventative service for example when a young person with multiple and complex needs leaves a children's home or foster care who is at heightened risk of homelessness (2, 69).

The British experience with HFW described here has also shown the risks that need to be effectively managed if HFW programmes are to function as effectively as possible. Clearly, a HFW service that can rapidly access the right sort of housing and good access to all the health, mental health, social work, addiction and other services that women experiencing homelessness with multiple and complex needs may require, will be more effective than HFW that face barriers to housing and those services. Equally, effective safeguarding where there is an ongoing risk to women using HFW requires good working relationships with the Police and wider criminal justice system and, if this is not present, safeguarding may become difficult.

HFW is also not a perfect service or programme model. In part, the issue here is that more evidence is needed on how to ensure HFW are structured in the best way possible to enable HFW programmes to work effectively as part of the kinds of integrated, preventative and housing led strategies that appear to be the best way of reducing homelessness (70). Failures will occur and limitations are present in the HFW model, which does have limits in terms of the complexity of need it can effectively handle, just as Housing First does (45) and which will experience failures in safeguarding. HFW programmes themselves also need to be properly financed on a sustainable basis, as insufficient and insecure funding will undermine an intervention which is designed to be open ended and will often be working with the women using it for years, rather than months.

Again, clearly differentiating between the needs of the younger, middle aged and (relatively) older women using HFW is difficult as all have multiple and complex issues requiring treatment and support. The specifics of working with middle aged and older women centre on the cumulative impacts of recurrent and sustained homelessness, i.e., their health, mental health, issues arising from addiction where present and the risks that they have had children from who they are separated will, on current evidence, all tend to increase. In essence, as is the case for Housing First in a broader sense, as the duration of homelessness experiences increases, the level of need for HFW is likely to rise in association (27), so that an older woman with longer experience of homelessness may well be more likely to need HFW.

In North America, there is a longstanding concern with ensuring that services are only accessed by those in the greatest need, i.e., only people who will definitely benefit from receiving support from a programme. This has led to arguments that Housing First is being used too late, i.e., by targeting people in the highest levels of need, Housing First risks becoming effectively a palliative intervention (44). HFW should not function only as a last resort service, which women with multiple and complex needs and longstanding experience of homelessness only access when their needs have become acute and they are approaching the end of their life, but should probably also be used in a more proactive, preventative framework for women at heightened risk of long term or repeated homelessness. There is a need for more evidence on how to facilitate a more preventative role, alongside a broader consideration of how to reduce the flow of women into situations of long-term and repeated homelessness through more effective preventative programmes. This said, HFW has the potential to create new and better programmes of services and enhance strategic responses for women with multiple and complex needs in situations of homelessness and for that reason alone, HFW should be subject to more testing and analysis (71–73).

## Data Availability

The raw data supporting the conclusions of this article will be made available by the authors, without undue reservation.

## Footnotes

^2^ https://housingfirsteurope.eu/wp-content/uploads/2022/12/HousingFirstWomen-2.pdf

^3^ https://ico.org.uk/for-organisations/uk-gdpr-guidance-and-resources/lawful-basis/a-guide-to-lawful-basis/special-category-data/

^4^ https://www.dahalliance.org.uk
